# The role of Neonatologist Performed Echocardiography in the assessment and management of neonatal shock

**DOI:** 10.1038/s41390-018-0081-1

**Published:** 2018-08-02

**Authors:** Willem P. de Boode, Robin van der Lee, Beate Horsberg Eriksen, Eirik Nestaas, Eugene Dempsey, Yogen Singh, Topun Austin, Afif El-Khuffash, on behalf of the European Special Interest Group ‘Neonatologist Performed Echocardiography’ (NPE), K. Bohlin, M. C. Bravo, C. R. Breatnach, M. Breindahl, A. M. Groves, S. Gupta, P. T. Levy, P. J. McNamara, Z. Molnar, S. R. Rogerson, C. C. Roehr, M. Savoia, U. Schubert, C. E. Schwarz, A. Sehgal, M. G. Slieker, C. Tissot, D. van Laere, B. van Overmeire, L. van Wyk

**Affiliations:** 1 Department of Neonatology, Radboud University Medical Center, Radboud Institute for Health Sciences, Amalia Children’s Hospital, Nijmegen, The Netherlands; 2 Department of Pediatrics, Møre and Romsdal Hospital Trust, Ålesund, Norway; 3 Institute of Clinical Medicine, Faculty of Medicine, University of, Oslo, Norway; 4 Department of Cardiology and Center for Cardiological Innovation, Oslo University Hospital, Rikshospitalet, Oslo, Norway; 5 Department of Paediatrics, Vestfold Hospital Trust, Tønsberg, Norway; 6 INFANT Centre, Cork University Maternity Hospital, University College, Cork, Ireland; 7 Addenbrooke’s Hospital, Cambridge University Hospitals NHS Foundation Trust, Cambridge, United Kingdom; 8 Department of Neonatology, Rosie Hospital, Cambridge University Hospitals NHS Foundation Trust, Cambridge, United Kingdom; 9 Department of Neonatology, The Rotunda Hospital, Dublin, Ireland; 10 Department of Pediatrics, The Royal College of Surgeons in Ireland, Dublin, Ireland; 11 Department of Neonatology, Karolinska University Hospital, Karolinska Institutet, Stockholm, Sweden; 12 Department of Neonatology, La Paz University Hospital, Madrid, Spain; 13 Department of Neonatology, The Rotunda Hospital, Dublin, Ireland; 14 Karolinska University Hospital, Karolinska Institutet, Stockholm, Sweden; 15 Division of Newborn Medicine, Mount Sinai Kravis Children’s Hospital, New York, NY USA; 16 University Hospital of North Tees, Durham University, Stockton-on-Tees, United Kingdom; 17 Department of Pediatrics, Washington University School of Medicine, Saint Louis, MO USA; 18 Department of Pediatrics, Goryeb Children’s Hospital, Morristown, NJ USA; 19 Departments of Pediatrics and Physiology, University of Toronto, Toronto, ON Canada; 20 John Radcliffe Hospital, Oxford, United Kingdom; 21 The Royal Women’s Hospital, Parkville, VIC Australia; 22 Department of Paediatrics, University of Oxford, John Radcliffe Hospital, Oxford, United Kingdom; 23 Azienda Ospedaliero-Universitaria S. Maria della Misericordia, Udine, Italy; 24 Department of Clinical Science, Intervention and Technology, Karolinska Institutet, Stockholm, Sweden; 25 Department of Neonatology, University Children’s Hospital of Tübingen, Tübingen, Germany; 26 Department of Pediatrics, Monash University, Melbourne, Australia; 27 Department of Paediatric Cardiology, Radboudumc Amalia Children’s Hospital, Nijmegen, The Netherlands; 28 Department of Pediatrics, Clinique des Grangettes, Chêne Bougeries, Switzerland; 29 Department of Pediatrics, Antwerp University Hospital UZA, Edegem, Belgium; 30 Department of Neonatology, University Hospital Brussels, Brussels, Belgium; 31 Department of Paediatrics & Child Health, University of Stellenbosch, Cape Town, South Africa

## Abstract

One of the major challenges of neonatal intensive care is the early detection and management of circulatory failure. Routine clinical assessment of the hemodynamic status of newborn infants is subjective and inaccurate, emphasizing the need for objective monitoring tools. An overview will be provided about the use of neonatologist-performed echocardiography (NPE) to assess cardiovascular compromise and guide hemodynamic management. Different techniques of central blood flow measurement, such as left and right ventricular output, superior vena cava flow, and descending aortic flow are reviewed focusing on methodology, validation, and available reference values. Recommendations are provided for individualized hemodynamic management guided by NPE.

## Introduction

The diagnosis and management of shock in the newborn infant presents many challenges to neonatologists. The determination of the adequacy of overall circulatory well-being remains predominantly subjective, and there are no validated clinical scoring systems available. Despite its many limitations, mean arterial blood pressure remains the most commonly used marker for circulatory compromise. Reliance on mean blood pressure values alone to determine circulatory well-being is an overly simplistic approach to a much more complex problem. A normal blood pressure does not equate to normal end-organ blood flow. There has been a recent move in other areas of medicine to incorporating multimodal monitoring in the management of complex clinical problems, such as neurocritical care. Multimodal monitoring provides the opportunity to overcome some of the shortcomings of each monitoring technique and ultimately achieves more accuracy in determining appropriate interventions. Echocardiography represents an objective tool to assist with the assessment of shock in the newborn infant. Functional echocardiography is rational and noninvasive, and may have a very important part to play in the overall assessment and management of newborn shock.

**Fig. 1:**
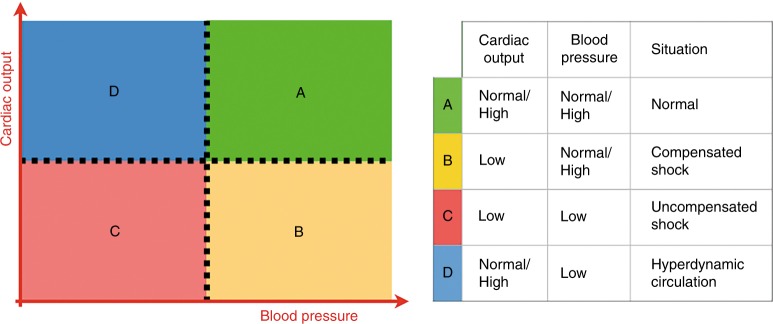
Identification of the stage of shock by simultaneous measurement of cardiac output and blood pressure

Shock is defined as a state of impaired cellular energy (ATP) synthesis when tissue oxygen delivery no longer satisfies tissue oxygen demand.^1^ In the first phase of shock, perfusion and oxygen delivery is maintained towards the so-called vital organs (heart, brain, and adrenal glands) by selective regional vasodilation in combination with vasoconstriction to non-essential tissues, such as muscles, skin, kidneys, and the splanchnic tissues. This compensated stage of shock is the result of neuroendocrine mechanisms. As the product of cardiac output (which falls) and systemic vascular resistance (which increases), blood pressure actually remains in the normal range in a compensated shock. When this redistribution fails, perfusion and oxygenation of the vital organs will become impaired, resulting in multi-organ dysfunction. It is in this phase of uncompensated shock that systemic hypotension might be expected. It should however be noted that—although controversial—data suggest that in very preterm infants the forebrain might be considered a non-vital organ, since the vasculature supplying the forebrain constrict in response to a decrease in perfusion.^2^ Moreover, cerebral autoregulation may be impaired in sick preterm infants, potentially resulting in (periods of) a pressure-passive cerebral perfusion.^3^ This all implies that in the vulnerable, very preterm infant a pressure-based approach might lead to an impaired perfusion and oxygenation of the cortex during the initial, compensated state of shock. The combination of low cardiac output with normal-to-high blood pressure suggests a compensated stage of shock, while low cardiac output in the presence of hypotension is indicative of an uncompensated stage of shock (see Fig. 1). A hyperdynamic circulation is characterized by a normal-to-high cardiac output in combination with hypotension. In transitional preterm physiology following birth, there is a physiological phenomenon that exists, where blood flow is normal with low blood pressure.

Echocardiography is one of the emerging technologies that can be used to measure cardiac output in critically ill newborn infants, especially since the clinical estimation of cardiac output is rather inaccurate.^4–6^ Moreover, Neonatologist Performed Echocardiography (NPE) can inform the clinician about possible underlying pathophysiologic mechanisms of circulatory failure and can be used to evaluate the efficacy of therapeutic interventions. The echocardiographic assessment of the hemodynamic status of the newborn has the potential to improve neonatal intensive care.^7–11^ In this review, we will discuss basic physiology regarding cardiac output and oxygen delivery, methodology, and validation of several techniques of central blood flow measurement (left and right ventricular output (RVO), superior vena cava flow (SVC flow), and descending aortic flow (DAo flow)), reference values, and recommendations for NPE-guided, individualized hemodynamic management.

## Oxygen delivery and cardiac output

The essential function of the circulatory system is the delivery of oxygen and nutrients to the tissues and transport of carbon dioxide and waste products to the excretory organs. Under normal circumstances oxygen delivery exceeds oxygen consumption.

Oxygen delivery is determined by serum hemoglobin concentration, arterial oxygen saturation, and cardiac output and is calculated by multiplying the arterial oxygen concentration (CaO_2_) by the cardiac output (CO). CaO_2_ is calculated as follows: (SaO_2_ (as gradient) × Hb (in mmol/L) × 0.98) + (PaO_2_ (in kPa) × 0.0004). As shown in this formula, the contribution of dissolved oxygen to the total arterial oxygen content can be neglected. Oxygen consumption is influenced by the metabolic rate (sedation, pain, anxiety), thermogenesis (shivering, fever, catecholamines), and external work (work of breathing, sepsis, trauma, catabolism).^12^

Optimization of the oxygen balance can be accomplished by an increase in oxygen delivery (e.g., red blood cell transfusion, supplemental oxygen, or inotropes) or a decrease in oxygen demand (e.g., sedation, muscle relaxants, or antipyretics).

**Fig. 2:**
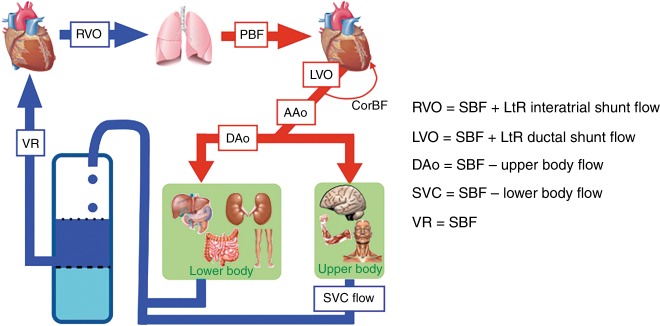
Central blood flow measurements. Different assessments of central blood flow are shown, such as LVO, RVO, SVC flow, and Dao flow. It should be noted that central blood flow does not always represent systemic blood flow in the presence of shunts. CorBF coronary blood flow, DAo descending aortic flow, LtR left-to-right, LVO left ventricular output, PBF pulmonary blood flow, RVO right ventricular output, SBF systemic blood flow, SVC superior vena cava flow, VR venous return

Cardiac output is not synonymous with systemic blood flow (SBF). Echocardiography can be used to estimate RVO, left ventricular output (LVO), DAo flow, and superior vena cava (SVC) flow (see Fig. 2). These central blood flows are not identical or interchangeable. In fact, the SBF, that is, the total blood flow perfusing the complete systemic vasculature and therefore supplying all tissues, would be the most informative. However, in the presence of fetal shunts, measurements of CO will not reflect the SBF. A left-to-right (LtR) ductal shunt will increase LVO and thereby overestimate the amount of blood that actually reaches the systemic circulation, since LVO = SBF + ductal LtR flow.^13^ A LtR shunt through a patent foramen ovale will increase RVO and thereby overestimate the systemic venous return, because RVO = SBF + interatrial LtR shunt flow.^14,15^ Similarly, shunts due to congenital heart defects, for example, a LtR shunt through a ventricular septal defect or a LtR shunt at the atrial level, will increase RVO, thereby overestimating SBF. DAo flow represents LVO minus upper body blood flow and coronary blood flow.

Given the potential influence of a shunt on the obtained value of cardiac output, it is imperative to also perform NPE when utilizing other techniques for cardiac output assessment (e.g., indicator dilution, thoracic electrical bioimpedance). NPE will provide information about the existence of such shunt. Those shunts cannot always be assessed with these other modalities. Knowledge of the presence of those shunts is vital for the correct interpretation of the obtained cardiac output values, unless these shunts are detected by the used technology itself.^16^

## Basic principles of echocardiographic-derived cardiac output assessment

Doppler technology can be used to measure blood flow velocity, as the moving red blood cells will produce a so-called Doppler shift. Velocity–time waveforms are then produced by spectral analysis of this frequency shift. The velocity–time integral (VTI), or the calculated area under the velocity–time curve, represents the stroke distance, that is, the distance that a column of blood will travel during one heart cycle. When the cross-sectional area (CSA) of this blood column is known, one can calculate stroke volume (SV = VTI x CSA). Hence, echocardiographic assessment of blood flow can be performed by multiplying the CSA (CSA = π (diameter/2)^2^) of the outflow tract by the VTI of blood flow across the outflow tract and the heart rate (HR), applying the equation below^13,17–19^

1 $$	{\mathrm{Blood}}\,{\mathrm{flow}}\,\left( {\mathrm{{mL/kg/min}}} \right) \\ 	= \frac{{\left( {\left( {\pi \times \left( {D{\mathrm{/}}2} \right)^2\,\left( {{\mathrm{cm}}^2} \right)} \right) \times {\mathrm{VTI}}\,\left( {\mathrm{cm}} \right) \times {\mathrm{HR}}\,\left( {\mathrm{bpm}} \right)} \right)}}{{{\mathrm{ }}{\mathrm{body}}\,{\mathrm{weight}}\,\left( {\mathrm{{kg}}} \right)}}.$$

The formula used to calculate the CSA is in principle only applicable in an ideal round-shaped vessel. In infants, it is advisable to take body size into consideration and index blood flow by body surface area (BSA) or by body weight.^19^ Given the inaccuracy of the calculation of BSA in newborns we advise to index cardiac output by body weight. In addition, the measurement of stroke distance occurs in the center of the vessel, where blood travels at highest velocity. This does not take into account the slower traveling blood around the periphery of the vessel close to the vessel wall. This means that using this method to measure cardiac output invariably results in an overestimation of the true value.^20^

An important principle in flow assessment is to record Doppler sampling of velocities at the same site where diameter measurements are performed. Pulsed-wave (PW) Doppler and not continuous-wave (CW) Doppler is advised for this purpose. Assessment of blood flow velocity should be avoided in areas just distal to a (relative) stenosis. Furthermore, care should be taken to minimize the angle between the blood flow direction and the Doppler beam to avoid an underestimation of the VTI. An angle of insonation up to 20° will result in a maximum reduction in blood flow velocity of 6%. One should be aware that in the obtained 2D image the out-of-plane angle error is not always observed, so the error might be even larger than measured in the 2D image. A standardized methodology will improve reproducibility and is able to detect changes over time.

The intraobserver variability of echocardiographic SBF measurements is rather high and reported as 12, 22, 17, and 14% for RVO, LVO, SVC flow, and DAo flow, respectively.^21–25^ The inter-observer variability is even higher. This disappointing repeatability is probably related to: (1) the difficulty in the exact measurement of the CSA, which is derived from measuring vessel diameter to obtain the radius, which is subsequently squared; (2) the assumption of a perfect circular form of the outflow tract; (3) the inaccuracy in the assessment of the VTI; (4) the assumption of laminar blood flow, and (5) the error secondary to the angle of insonation. It should be noted that there is also an intrinsic biological variation in stroke volume secondary to cardiopulmonary interaction, resulting in the highest level of SVC flow during inspiration and highest LVO at end expiration.^26^ To minimize the influence of respiration on stroke volume measurements, it is advised to average VTI calculations of at least three to five heart cycles.

Reference values for diameters of the right and left ventricular outflow tract and superior vena cava (SVC) in preterm infants have been published that could be used to check diameter measurements and prevent the use of outliers.^27^

### Left ventricular output

#### Methodology

**Fig. 3:**
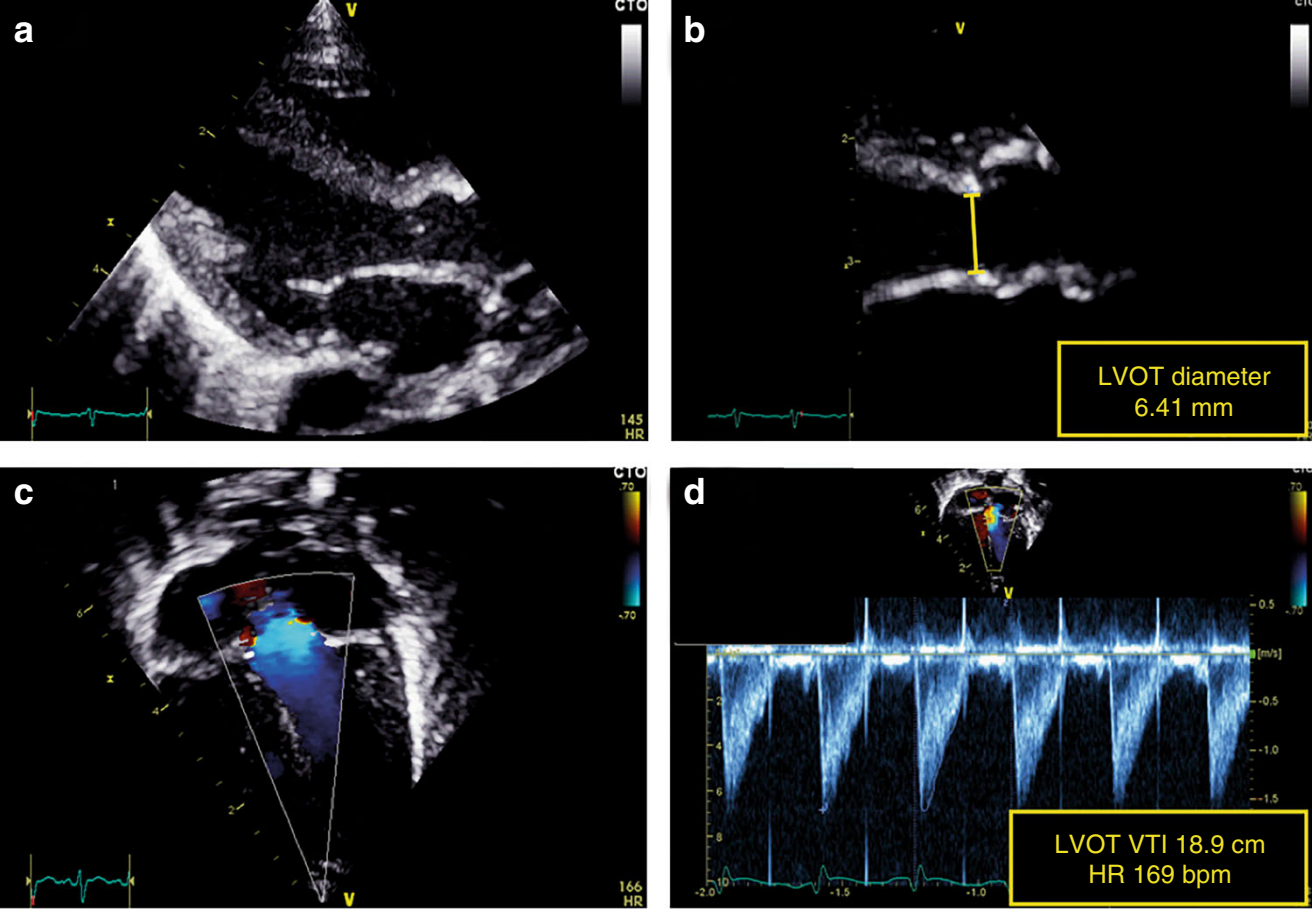
Assessment of left ventricular output (LVO). LV outflow tract diameter is measured from the parasternal long-axis view (**a**). The diameter measurement should be performed at the hinge points of the aortic valve at end-systole (**b**). The velocity time integral (VTI) in the LV outflow tract should be assessed in the apical five-chamber view or apical long-axis view using pulsed-wave Doppler with the sample volume just below the aortic valve (**c**). The pulsed wave Doppler recording is paused to obtain a smooth VTI envelope for exact tracing of the signal (**d**)

LVO equals the SBF in the absence of ductal shunting. LVO tract diameter is usually measured from the parasternal long-axis view atend-systole (see Fig. 3).^28^ Variations in diameter location (sub-valvular region, hinge points of the aortic valve, or start of the ascending aorta) and method of diameter measurement (2D or M mode, leading edge or trailing edge) occurs in the literature.^29^ Variations also exist in VTI acquisition, both in Doppler method (PW or CW) and transducer position (apical, subcostal, or suprasternal). We recommend that diameter measurements should be performed at the hinge points of the aortic valve at end-systole. VTI in the LV outflow tract should be assessed by PW Doppler in the apical five-chamber view or apical long-axis view with the sample volume just below the aortic valve.^30^ We recommend 2D freezing during PW Doppler recordings to obtain a smooth VTI envelope for more exact tracing of the signal.

#### Validation

The assessment of LVO by echocardiography and magnetic resonance imaging (MRI) correlate strongly.^31^ However, LVO only equals SBF in the absence of a ductal (LtR) shunt. Validation studies in newborn infants, comparing LVO assessed with transthoracic echocardiography with a gold standard reference method, are rather scarce. In a recent study, LVO measurement was compared between echocardiography and phase contrast MRI (PC-MRI) in 47 term and preterm newborn infants and showed a mean bias of −9.6 mL/kg/min and limits of agreement (LOA, that is, ±1.96 × SD) of ±70 mL/kg/min resulting in a bias percentage (mean bias/mean LVO) of −3.8% and an error percentage (LOA/mean LVO) of ±28.2%.^31^ Validation studies in a pediatric population comparing echocardiography with other technologies, such as dye dilution, Fick, and thermodilution technologies for cardiac output measurement, showed a bias percentage of <10%, but with a rather large range (−37% to +16%) and an error percentage of ±30%.^23^

### Right ventricular output

#### Methodology

**Fig. 4:**
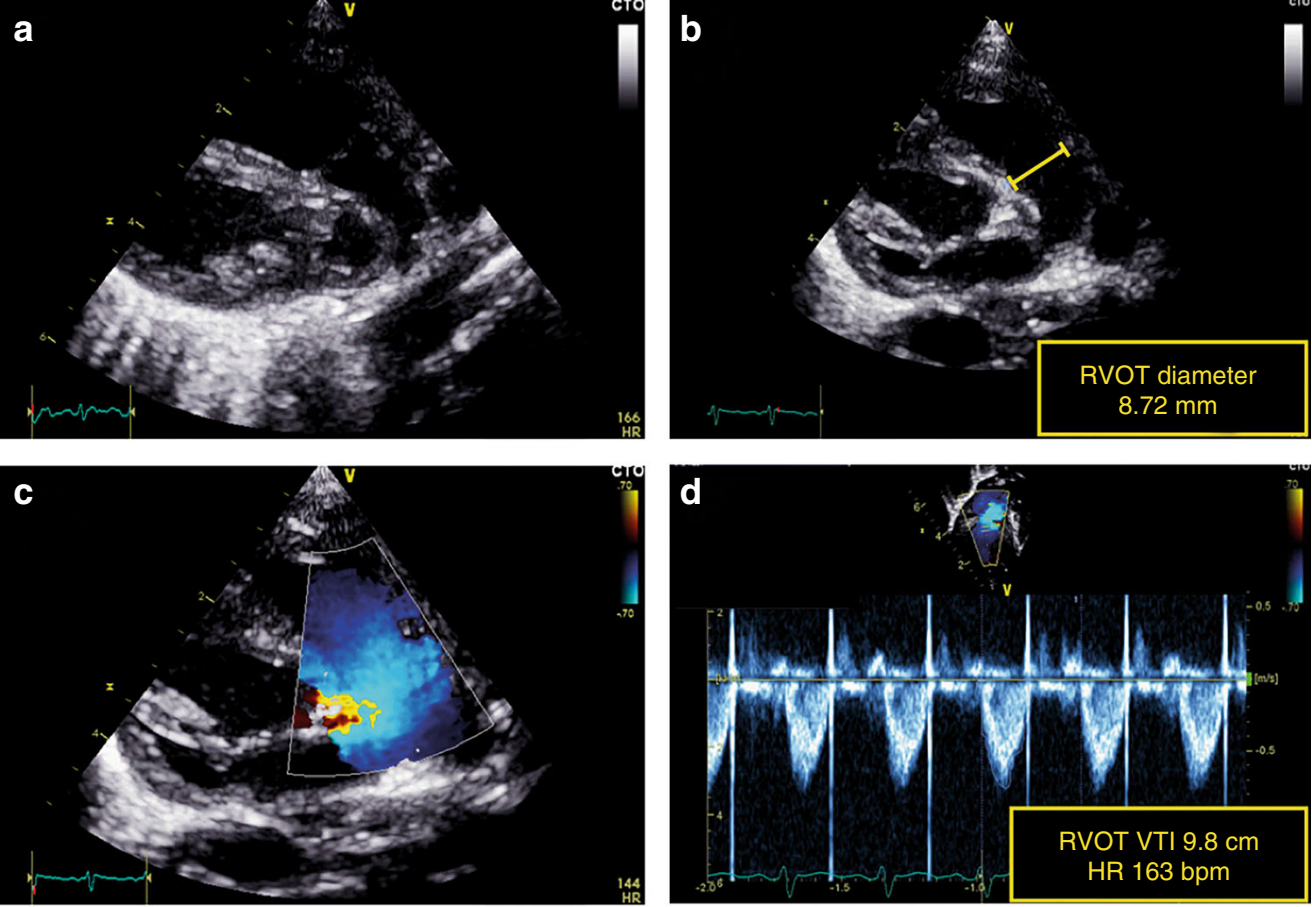
Assessment of right ventricular output (RVO). RV outflow tract diameter is measured from the tilted parasternal long-axis view (**a**). The diameter measurement should be performed at the pulmonary valve insertion (**b**). The velocity–time integral (VTI) is acquired in the same view or in the parasternal short-axis view, depending on angle orientation (**c**). The pulsed wave Doppler recording is paused to obtain a smooth VTI envelope for exact tracing of the signal (**d**)

RVO can be obtained using the same principles as for LVO. RVO reflects systemic venous return in the absence of fetal shunts. RV outflow tract diameter is usually measured from the tilted parasternal long-axis view at the pulmonary valve insertion (see Fig. 4).^13^ The VTI is acquired in the same view or in the parasternal short-axis view depending on angle orientation.^22^ Of note, in the presence of a patent ductus arteriosus, it may be difficult to obtain an accurate estimation of RVO because the true VTI can be very obscured by LtR shunting.

#### Validation

To our knowledge, no data are available comparing echocardiographic-derived RVO with a gold standard reference method.

### SVC flow

#### Methodology

SVC flow reflects the blood returning from the brain and upper part of the body to the heart. SVC flow has therefore been proposed as a surrogate measure of systemic, and especially cerebral blood flow by the following calculation:^21^

2 $$	{\mathrm{SVC}}\,{\mathrm{flow}}\,\left( {{\mathrm{mL}}{\mathrm{/}}{\mathrm{kg}}{\mathrm{/}}{\mathrm{min}}} \right) \\ 	= \frac{{\left( {\left( {\pi \times \left( {{\mathrm{mean}}\,{\mathrm{SVC}}\,D{\mathrm{/}}2} \right)^2\left( {{\mathrm{cm}}^2} \right)} \right) \times {\mathrm{VTI}}\,\left( {{\mathrm{cm}}} \right) \times {\mathrm{HR}}\,\left( {{\mathrm{bpm}}} \right)} \right)}}{{{\mathrm{body}}\,{\mathrm{weight}}\,\left( {{\mathrm{kg}}} \right)}}.$$

In a strict sense, SVC flow does not represent cardiac output, but only partial cardiac input. In the original paper describing SVC flow in the neonate, Kluckow and Evans^21^ advised pulsed Doppler recordings for VTI tracings from a low subcostal view and diameter measurements in a sagittally rotated high parasternal view. The SVC is a vein and therefore not ideally rounded in shape. It will also vary in shape secondary to respiratory movements (intrathoracic pressure variation) and pulsatile movements during the heart cycle of the adjacent aorta. Diameter error can also introduce significant error in output values since the radius is squared in the calculation of cross-section area.

**Fig. 5:**
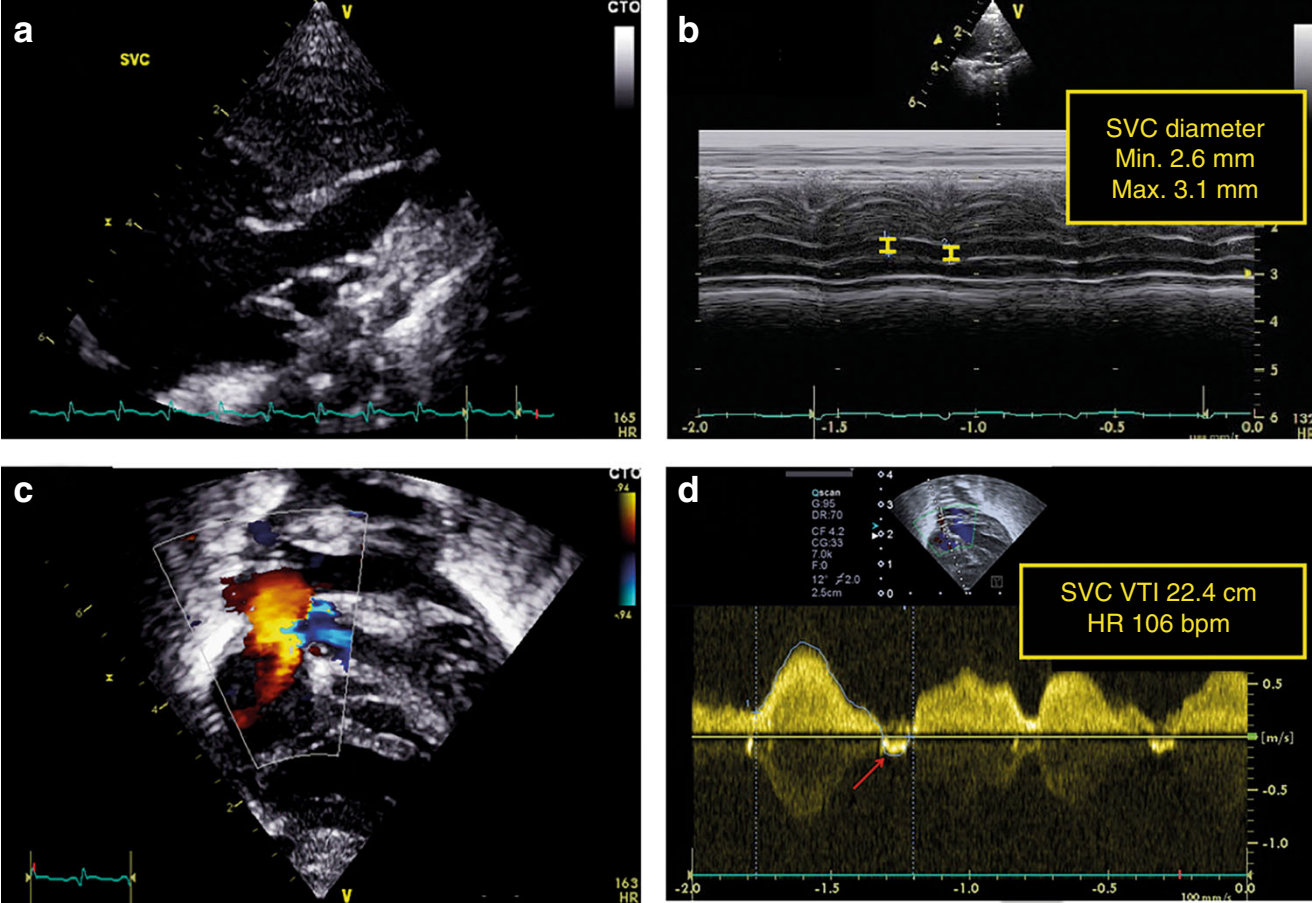
Assessment of superior vena cava (SVC) flow. The SVC diameter is measured at the entrance to the right atrium from the modified parasternal long-axis view (**a**). Both minimum and maximum diameters are measured through the heart cycle. Three to five consecutive heart cycles are analyzed with the average regarded as the mean SVC diameter (**b**). The subcostal view is used to assess SVC flow velocity (**c**). It is advised to average VTI tracings from eight to ten heart cycles to minimize variations secondary to respiration. Negative blood flow velocities (A-wave and sometimes a late-systolic negative wave) should be subtracted for a reliable SVC flow estimation (**d**)

There are several approaches that have recently been employed to overcome problems with obtaining reliable SVC diameters. A modified parasternal long-axis view has been used to assess SVC diameter. It is important to zoom and focus on the SVC at the entrance in the right atrium (see Fig. 5). Both minimum and maximum diameters are measured through the heart cycle. With this approach, three to five consecutive heart cycles are analyzed with the average regarded as the mean SVC diameter. In addition to the parasternal long-axis view, direct assessment of the SVC area in a short-axis view at the level of the right pulmonary artery resulted in an increase in accuracy and repeatability.^31^ In other studies, the suprasternal view was suggested to assess SVC flow velocity as an alternative to the subcostal view to minimize discomfort and erroneous blood flow velocity measurement due to abdominal movement.^32,33^ It is advised to average VTI tracings from eight to ten heart cycles to minimize variations secondary to respiration. Negative blood flow velocities (A-wave and sometimes a late-systolic negative wave) should be subtracted for a reliable SVC flow estimation.

SVC flow may be applied as a substitute for cardiac output in assessing systemic flow when fetal shunts are present, but awareness of the shortcomings of the method is important in the interpretation.

#### Validation

Reference values in term and preterm infants have been published, but with considerable variations,^21,24,25^ and especially the SVC diameter seems prone to measurement error.^25^ In a validation study of blood flow in 23 newborn infants, assessment of SVC flow by echocardiography and PC-MRI showed poor correlation.^31^ This study specifically showed a mean bias of −13.7 mL/kg/min and LOA of ±75 mL/kg/min, representing a bias and error percentage of −13.5 and 73.7%, respectively, when calculating SVC flow using SVC diameter. Of note, the validity of this study has been questioned.^34,35^ Direct assessment of the SVC area from an axial view in combination with a 50% reduction in measured stroke distance to compensate for structural overestimation resulted in just a minor improvement in accuracy and precision (bias percentage of 2.6% and an error percentage of ±55%).^31^

A modified approach to SVC assessment that utilizes both the measurement of SVC flow from the suprasternal view and the tracing of the SVC area from a short-axis view at the level of the right pulmonary artery has recently been shown to improve reliability of SVC flow quantification in neonates.^36^ This modified approach of SVC assessment was compared between echocardiography and PC-MRI and showed improved accuracy with a bias percentage of 17.7% and an error percentage of 36.9%.^36^ Although this approach resulted in an improved accuracy, the error percentage is still disappointingly high.

Several studies have shown an association between low SVC flow in the first 24 h and intraventricular hemorrhagevand/or neonatal death in preterm infants,^37–39^ whereas others found no association.^40,41^ Conflicting results in reference values and clinical utility renders SVC flow to be of limited applicability.^31^

### Descending aortic flow

#### Methodology

Blood flow in the DAo, below the level of the ductus arteriosus, equals the SBF to the lower part of the body. This parameter has been assessed in preterm infants and children.^24,42,43^ Due to larger inconsistencies in DAo diameter measurements compared to Doppler flow velocities, VTI measurements alone have been proposed as a marker of lower body SBF.^24^ In the presence of a persistent ductus arteriosus, the pattern of DAo flow can also provide information on the magnitude of ductal shunting.^44,45^

**Fig. 6:**
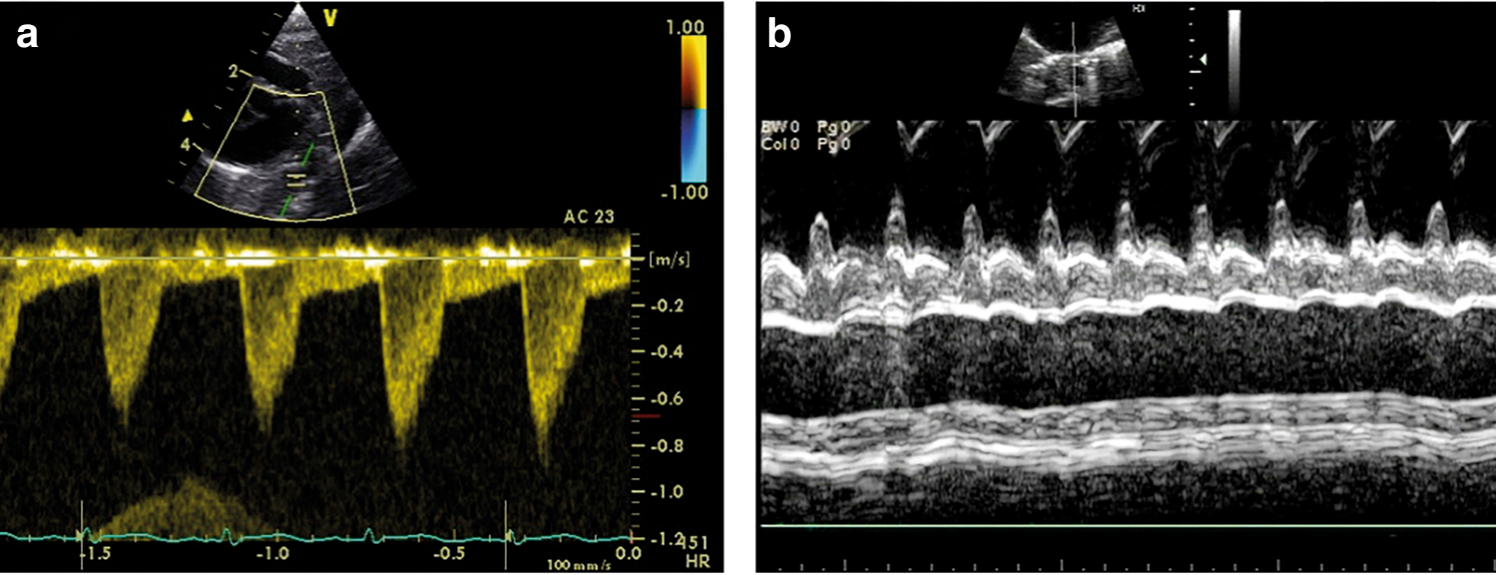
Assessment of descending aorta (DAo) flow. The velocity–time integral (VTI) of DAo flow can be assessed both from a high parasternal or low subcostal sagittal view using pulse-wave Doppler with a minimum angle of insonation (**a**). In the presence of any retrograde diastolic aortic blood flow, the DAo flow should be corrected by deducting the retrograde VTI from the antegrade VTI. The diameter of the DAo is measured in the short axis parasternal axis view, as close as possible to the plane of the aortic valve (**b**)

The VTI of DAo flow can be assessed both from a high parasternal or low subcostal sagittal view using PW Doppler with a minimum angle of insonation. In the presence of any retrograde diastolic aortic blood flow the DAo flow should be corrected by deducting the retrograde VTI from the antegrade VTI. This approach may produce artificially low values for total DAo flow, since the retrograde component of flow tends to be non-laminar, and therefore over-estimated when inferring volume of flow from maximum velocity. The DAo diameter is measured in the parasternal short-axis view, as close as possible to the plane of the aortic valve (see Fig. 6).^24^ DAo flow is calculated by the following formula:

3 $$	{\mathrm{DAo}}\,{\mathrm{flow}}\left( {{\mathrm{mL}}{\mathrm{/}}{\mathrm{kg}}{\mathrm{/}}{\mathrm{min}}} \right) \\ 	{= \frac{{\left( {\left( {\pi \times \left( {{\mathrm{mean}}\,{\mathrm{DAo}}\,D{\mathrm{/}}2} \right)^2\left( {{\mathrm{cm}}^2} \right)} \right) \times \left( {{\mathrm{antegrade}} - {\mathrm{retrograde}}} \right)\,{\mathrm{VTI}}\,\left( {{\mathrm{cm}}} \right) \times {\mathrm{HR}}\,\left( {{\mathrm{bpm}}} \right)} \right)}}{{{\mathrm{body}}\,{\mathrm{weight}}\,\left( {{\mathrm{kg}}} \right)}}.}$$

#### Validation

One study has assessed the intraobserver and inter-observer variability of DAo diameter and stroke distance measurement and found very poor repeatability for the two measurements in preterm infants <31 weeks’ gestation.^24^ To our knowledge, no studies have been published comparing echocardiographic-derived DAo flow with a gold standard reference method.

## Reference values for cardiac output

The ventricular output in normal preterm neonates without transductal and interatrial shunting is 150–300 mL/kg/min.^13,46–48^ Reference values for SBF in term and preterm infants are shown in Table 1.^13,29,37,49–51^

**Table 1:** Reference values blood flow measurements in mean (SD) mL/kg/min ^12,28,36,38–50^

	Postnatal age			
	3–9 h	24 h	Day 2	Days 7–14
***RVO***				
Preterm		260 (90)	270 (90)	430 (100)
Term		255 (60)		
***LVO***				
Preterm		240 (60)	260 (60)	400 (75)
Term		220 (60)		
***SVC flow***				
Preterm	60 (25)	80 (20)	90 (25)	90 (30)
Term	75 (25)	95 (30)	100 (30)	

*RVO* right ventricular output, *LVO* left ventricular output, *SVC* superior vena cava

In 28 newborn infants with proven ductal closure in a stable condition at a median postnatal age of 10 days the mean (SD) value of LVO, RVO, SVC flow, and DAo flow was 222 (45.8), 219 (46.9), 95 (27), and 126 (32.1) mL/kg/min, respectively.^52^

It is not possible to define an absolute minimum value of SBF since cardiac output is just one of the determining parameters of the oxygen balance (oxygen delivery in relation to oxygen consumption). When the basal metabolic rate and oxygen need is low, that is, during therapeutic hypothermia and under general anesthesia, a relatively low SBF might suffice and will not be associated with tissue injury. However, studies have shown an association with increased morbidity and mortality when ventricular output is <150 mL/kg/min or SVC flow is <30 mL/kg/min at a postnatal age of 5 h or <40–45 mL/kg/min afterwards.^13,37–39,53–56^

Risk factors for a low SBF are low gestational age, severe respiratory distress syndrome, mechanical ventilation with a high mean airway pressure, and a large PDA.^51,54^

In clinical practice however, the absolute value of cardiac output might be of less importance than the spectrum in which the measured values reside. The level of cardiac output can be categorized as low, normal, or high. Combining the information about the levels of blood pressure and cardiac output together with echocardiographic information about the presence of shunts, myocardial function, pulmonary pressure, and volume status enables the clinician to comprehensively assess the hemodynamic status of a newborn infant and to estimate underlying pathophysiology. Provided that adequate therapeutic interventions are initiated, this will have the potential to prevent tissue injury and improve outcome.

## Assessment of volume status

Adequate intravascular volume is needed to generate sufficient preload to the heart. Volume expansion is often used as first-line therapy for hemodynamic compromise in neonates.^57^ In the case of true hypovolemia fluid resuscitation is important; however, excessive fluid administration is associated with an increase in morbidity and mortality.^58,59^ Potential mechanisms for these adverse effects are that volume overload will result in tissue edema. Moreover, data suggest that an increased release of natriuretic peptides in response to elevated cardiac filling pressures is associated with injury to the endothelial glycocalyx leading to an increase in endothelial permeability.^60,61^ It would be very helpful if the response to and the need for volume expansion could be predicted. Volume responsiveness is defined as an increase in stroke volume (5–10%) secondary to a fluid bolus.^62^ However, a fluid responder does not automatically imply a hypovolemic state with a need of volume expansion. Clinical (static) hemodynamic parameters, such as heart rate and blood pressure, cannot be used to predict fluid responsiveness reliably in newborn infants.^6,63^ Dynamic indices of fluid responsiveness, such as arterial blood pressure variation and stroke volume variation secondary to heart–lung interactions, have been studied in adults and older children under specific circumstances and have shown promising predictive values. However, these methods are not applicable in daily practice in intensive care.^64–67^

To date, no studies have assessed the predictive value of dynamic hemodynamic variables for fluid responsiveness in preterm infants. The prediction of volume responsiveness, for example, by analyzing arterial blood pressure variation secondary to cardiorespiratory interaction, is hampered in neonates, because of a phenomenon called physiological aliasing as a result of a relatively low HR/respiratory rate ratio.^68^

In the absence of validated, objective predictive hemodynamic parameters of volume responsiveness in newborn infants, only subjective and unreliable echocardiographic markers of hypovolemia are available, such as left ventricular end-diastolic diameter, left atrial diameter, LA/Ao ratio, and diameter and collapsibility of the inferior vena cava.^63,69,70^

To assess IVC filling place the ultrasound transducer in the midline, just below the xiphisternum, and in the sagittal plane.^71^ The probe marker should be pointing towards the head, so that the heart appears just visible on the right of the screen. The IVC can be seen coursing through the liver. A normally filled IVC will have some pulsation with the cardiac cycle and respiratory motion. An under-filled IVC will be barely visible or collapse entirely on inspiration. An over-filled IVC will appear large, and minimally pulsatile. But beware of the ventilated infant, especially those on high frequency oscillatory ventilation; high intrathoracic pressure can effectively tamponade venous return at the level of the IVC, making the IVC appear well-filled, when the cardiac chambers themselves are under-filled. Therefore, when assessing preload status always also examine the intracardiac filling. This is often most easily accomplished from the subcostal view. This is convenient as it can follow directly on from the sagittal subcostal view used for IVC assessment.

However, the lack of reliable indicators has led to the estimation of the volume status by subjective interpretation of volume loading of the heart (“eyeballing”), which cannot be advised given its inaccuracy. Overall, NPE is not the best tool to assess intravascular volume in newborn infants.

## Discussion

The use of NPE for assessment of SBF is encouraged since it provides in combination with blood pressure measurement essential information about the hemodynamic status of the newborn infant. It enables a targeted hemodynamic management approach that takes into account the underlying pathophysiologic mechanisms of circulatory failure in the individual patient.

Monitoring cardiac output is only one of the pivotal steps in our effort to improve outcome in critically ill newborn infants. A “normal” level of SBF does not automatically imply adequate perfusion of all tissues. Cardiac output assessment will only provide information on central blood flow, and not on regional (organ) blood flow. Near-infrared spectroscopy has been introduced in clinical practice to monitor cerebral hemodynamics and oxygenation, and can also be used to assess renal and splanchnic perfusion.^72–75^ Increased regional fractional oxygen extraction might reflect redistribution phenomena to preserve perfusion of the vital organs. Moreover, simultaneous assessment and coherence analysis of arterial blood pressure and regional cerebral oxygen saturation (rScO_2_) provides information on cerebral autoregulatory capacity.^76–78^ One should however be aware that rScO_2_ will only reflect cerebral blood flow when the cerebral metabolic rate of oxygen, arterial oxygen saturation, hemoglobin concentration, and arterial partial pressure of carbon dioxide are expected to be stable and in the absence of artifacts. Assessment of regional perfusion will augment the hemodynamic assessment upon which a tailored management can be founded.

**Fig. 7:**
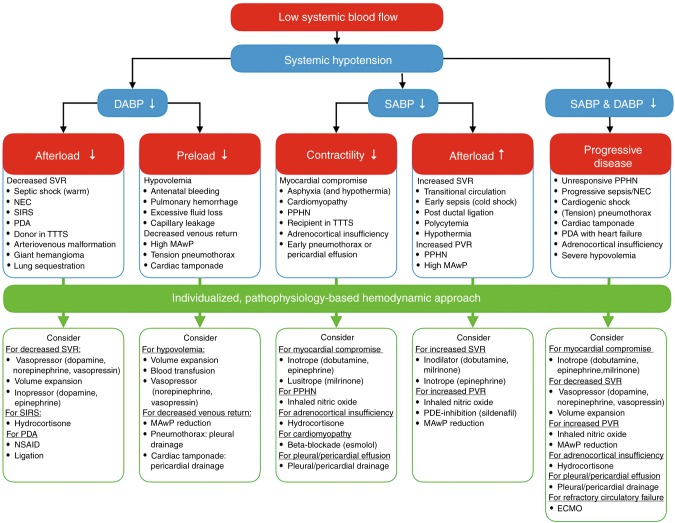
Algorithm for an individualized, pathophysiology-based approach towards a low cardiac output state in newborn infants. Please note that there may be an overlap between underlying pathophysiological mechanisms and that they are not necessarily mutually exclusive. ECMO extracorporeal membrane oxygenation, MAwP mean airway pressure, NSAID non-steroidal anti-inflammatory drug, PPHN persistent pulmonary hypertension of the newborn, PDA patent ductus arteriosus, PDE phosphodiesterase, PVR pulmonary vascular resistance, SIRS systemic inflammatory response syndrome, SVR systemic vascular resistance, TTTS twin-to-twin transfusion syndrome

**Fig. 8:**
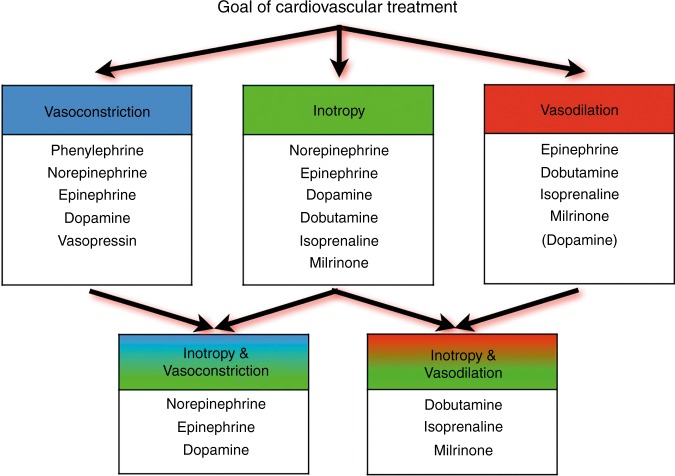
Potential choices of cardiovascular drugs based upon the preferred hemodynamic effect (cardiovascular drugs presented in a random, non-prioritized order)

**Fig. 9:**
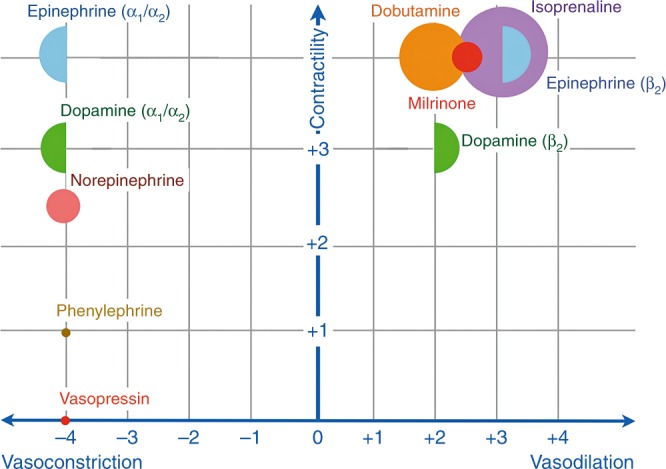
Presumed effects of commonly used cardiovascular drugs in neonatal intensive care. *X*-axis (effect on vascular tone): the more to the right, the more vasodilatory effects; the more to the left, the more vasoconstrictory effects. *Y*-axis (inotropic properties): the higher on the *Y*-axis, the more inotropic characteristics. The larger the size of the (semi-)circle, the more chronotropic effects. It should be noted that the effect on vascular tone depends on the used dosage that determines which adrenergic receptors are activated (e.g., dopamine and epinephrine). Based on refs. ^77, 78^

Comprehensive hemodynamic monitoring followed by adequate interpretation of the obtained parameters together with an individualized, targeted approach is essential to decrease mortality and morbidity. Elsayed et al.^79^ reported a retrospective study with faster clinical recovery in hemodynamically unstable newborns after implementation of comprehensive hemodynamic monitoring, including the use of NPE. After diagnosing the underlying pathophysiologic mechanisms of hemodynamic compromise, one should choose the appropriate therapeutic intervention. This means, for example, inotropes in the presence of myocardial dysfunction, vasopressors in a state of vasoplegia, or vasodilators to treat increased afterload. Figure 7 shows an algorithm for an individualized, pathophysiology-based approach towards a low cardiac output state in combination with systemic hypotension in newborn infants. As mentioned before, it is also possible to have low cardiac output without systemic hypotension, depending on systemic vascular resistance. Figures 8 and 9 can be used as guidance for the choice of treatment.^80,81^ A recent review outlines management strategies based on disease pathophysiology, incorporating SBF measurements.^82^ The hemodynamic effects of the initiated treatment regimen should be monitored, evaluated, and adjusted if needed.

There are some limitations with the utilization of echocardiography in neonatal intensive care. Training is required before NPE can be used safely for hemodynamic assessment and therapy guidance.^83^ Furthermore, the assessment takes some time and may lead to clinical instability. Another limitation is the rather high inter-rater variability. In addition, echocardiography is not a continuous monitoring tool and therefore not ideal for trend monitoring. Several international bodies are addressing training and certification in functional echocardiography in newborn care, which will provide an important role in ensuring that this assessment tool is correctly integrated. There is a paucity of good prospective (randomized) studies demonstrating improved outcomes of conventional monitoring vs. more comprehensive hemodynamic monitoring. Real-time interpretation and integration into individualized targeted therapy, along with other assessment tools, remains the next challenge.

## Conclusion

Neonatal hemodynamic assessment is rather complex and should encompass more than solely monitoring HR, blood pressure, and other inaccurate clinical variables. NPE has the potential to play a pivotal role in a timely detection of cardiovascular failure, the initiation of an individualized treatment strategy, and monitoring the effects of therapeutic interventions. However, NPE will only improve outcome when it is performed by adequately trained professionals who are aware of both its benefits and limitations, are capable of a correct interpretation of acquired information, and have knowledge about underlying pathophysiology and appropriate treatment strategies.

## APPENDIX

**European Special Interest Group ‘Neonatologist Performed Echocardiography’ (NPE), endorsed by the European Society for Paediatric Research (ESPR) and European Board of Neonatology (EBN)**

**de Boode W. P.** (chairman), Department of Neonatology, Radboud University Medical Center, Radboud Institute for Health Sciences, Amalia Children’s Hospital, Nijmegen, The Netherlands (willem.deboode@radboudumc.nl)

**Austin T.**, Department of Neonatology, Rosie Hospital, Cambridge University Hospitals NHS Foundation Trust, Cambridge, United Kingdom (topun.austin@addenbrookes.nhs.uk)

**Bohlin K.**, Department of Neonatology, Karolinska University Hospital, Karolinska Institutet, Stockholm, Sweden (kajsa.bohlin@ki.se)

**Bravo M. C.**, Department of Neonatology, La Paz University Hospital, Madrid, Spain (mcarmen.bravo@salud.madrid.org)

**Breatnach C. R.**, Department of Neonatology, The Rotunda Hospital, Dublin, Ireland (colm.breatnach@gmail.com)

**Breindahl M.**, Karolinska University Hospital, Karolinska Institutet, Stockholm, Sweden (morten.breindahl@sll.se)

**Dempsey E.**, INFANT Centre, Cork University Maternity Hospital, University College Cork, Ireland (g.dempsey@ucc.ie)

**El-Khuffash A.**, Department of Neonatology, The Rotunda Hospital, Dublin, Ireland; Department of Pediatrics, The Royal College of Surgeons in Ireland, Dublin, Ireland (afifelkhuffash@rcsi.ie)

**Groves A. M.**, Division of Newborn Medicine, Mount Sinai Kravis Children’s Hospital, New York, NY, USA (alan.groves@mssm.edu)

**Gupta S.**, University Hospital of North Tees, Durham University, Stockton-on-Tees, United Kingdom (samir.gupta@nth.nhs.uk)

**Horsberg Eriksen B.**, Department of Pediatrics, Møre and Romsdal Hospital Trust, Ålesund, Norway (beate.eriksen@me.com)

**Levy P. T.**, Department of Pediatrics, Washington University School of Medicine, Saint Louis, MO, USA; Department of Pediatrics, Goryeb Children’s Hospital, Morristown, NJ, USA (Levy_P@kids.wustl.edu)

**McNamara P. J.**, Departments of Pediatrics and Physiology, University of Toronto, Toronto, ON, Canada (patrick.mcnamara@sickkids.ca)

**Molnar Z.**, John Radcliffe Hospital, Oxford, United Kingdom (zoltan.Molnar@ouh.nhs.uk)

**Nestaas E.**, Institute of Clinical Medicine, Faculty of Medicine, University of Oslo, Norway; Department of Cardiology and Center for Cardiological Innovation, Oslo University Hospital, Rikshospitalet, Oslo, Norway; Department of Paediatrics, Vestfold Hospital Trust, Tønsberg, Norway (nestaas@hotmail.com)

**Rogerson S. R.**, The Royal Women's Hospital, Parkville, VIC, Australia (sheryle.Rogerson@thewomens.org.au)

**Roehr C. C.**, Department of Paediatrics, University of Oxford, John Radcliffe Hospital, Oxford, United Kingdom (charles.roehr@paediatrics.ox.ac.uk)

**Savoia M.**, Azienda Ospedaliero-Universitaria S. Maria della Misericordia, Udine, Italy (marilena.savoia@gmail.com)

**Schubert U.**, Department of Clinical Science, Intervention and Technology, Karolinska Institutet, Stockholm, Sweden (ulfschubert@gmx.de)

**Schwarz C. E.**, Department of Neonatology, University Children’s Hospital of Tübingen, Tübingen, Germany (c.schwarz@med.uni-tuebingen.de)

**Sehgal A.**, Department of Pediatrics, Monash University, Melbourne, Australia (arvind.sehgal@monash.edu)

**Singh Y.**, Addenbrooke's Hospital, Cambridge University Hospitals NHS Foundation Trust, Cambridge, United Kingdom (yogen.Singh@nhs.net)

**Slieker M. G.**, Department of Paediatric Cardiology, Radboudumc Amalia Children’s Hospital, Nijmegen, The Netherlands (Martijn.Slieker@radboudumc.nl)

**Tissot C.**, Department of Pediatrics, Clinique des Grangettes, Chêne Bougeries, Switzerland (cecile.tissot@hotmail.com)

**van der Lee R.**, Department of Neonatology, Radboud University Medical Center, Radboud Institute for Health Sciences, Amalia Children’s Hospital, Nijmegen, The Netherlands (Robin.vanderLee@radboudumc.nl)

**van Laere D.**, Department of Pediatrics, Antwerp University Hospital UZA, Edegem, Belgium (david.VanLaere@uza.be)

**van Overmeire B.**, Department of Neonatology, University Hospital Brussels, Brussels, Belgium (bart.van.overmeire@erasme.ulb.ac.be)

**van Wyk L.**, Department of Paediatrics & Child Health, University of Stellenbosch, Cape Town, South Africa (lizelle@sun.ac.za)
